# Supplementary material for: Venomix: a simple bioinformatic pipeline for identifying and characterizing toxin gene candidates from transcriptomic data
Source: PeerJ. 2018 Jul 31;6:e5361. doi: 10.7717/peerj.5361 (PMC6074769; doi:10.7717/peerj.5361)
Supplement: Supplemental Information 3 [file peerj-06-5361-s003.gz › FinalOutput_E-6/L-amino-acid_oxidase_1/finaltree.pdf]

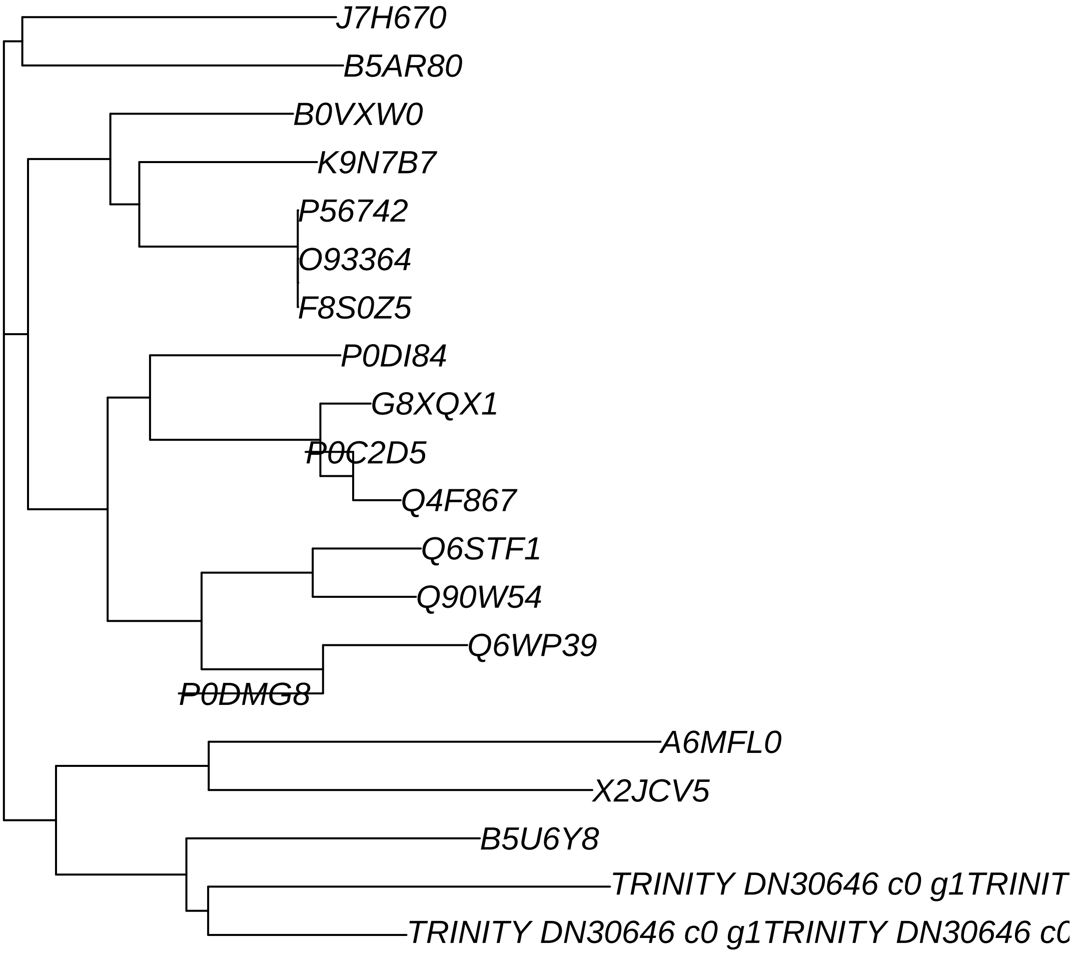
J7H670
B5AR80
B0VXW0
K9N7B7
P56742
O93364
F8S0Z5
P0DI84
G8XQX1
P0C2D5
Q4F867
Q6STF1
Q90W54
Q6WP39
P0DMG8
A6MFL0
X2JCV5
B5U6Y8
TRINITY DN30646 c0 g1TRINIT
TRINITY DN30646 c0 g1TRINITY DN30646 c0
